# Boron Nitride Interfacial Confinement of FeNi Layered Double Hydroxide Reconstruction for Durable Overall Water Splitting

**DOI:** 10.3390/nano16201280

**Published:** 2026-10-09

**Authors:** Jiahao Liu, Shitao Zhang, Rong He, Xiongfei Zhang, Yao Xiang

**Affiliations:** 1 Marist Brothers International School, Beijing 100103, China; 2 School of Chemical Sciences, University of Chinese Academy of Sciences, Beijing 100049, China; 3 Inner Mongolia Taixi Coal Group Co., Ltd., Alxa Left Banner, Alxa League 750306, Inner Mongolia, China

**Keywords:** interfacial corral, dynamic reconstruction, electronic modulation, water splitting, NiFe-LDH

## Abstract

Developing durable and highly active earth-abundant electrocatalysts is crucial for large-scale water splitting. While NiFe-based layered double hydroxides (LDHs) are promising for the oxygen evolution reaction (OER), their stability is often compromised by the irreversible structural degradation of their dynamically reconstructed active phase. Rationally controlling this reconstruction to simultaneously enhance intrinsic activity and structural stability remains a formidable challenge. Herein, we propose and demonstrate a strategy by engineering a crystalline boron nitride (BN) interface onto a FeNi LDH precatalyst. This single interface achieves a unique dual function. Theoretical calculations and operando spectroscopy reveals that the BN nanolayer first functions as a physical corral, effectively immobilizing the in situ formed FeNiOOH nanoclusters and preserving their high dispersion to ensure structural stability. Furthermore, this interface transcends the role of a passive shield; theoretical calculations confirm it also acts as a powerful electronic modulator, inducing interfacial charge transfer that results in a crucial upward shift in the Fe/Ni d-band center. This synergistic mechanism breaks the OER scaling limitations by lowering the energy barrier for the rate-determining step. As a result, the catalyst exhibits exceptional bifunctional performance, requiring low overpotentials of just 234 mV for OER and 98 mV for HER to achieve 10 mA cm^−2^. This enables a two-electrode electrolyzer to reach 10 mA cm^−2^ at a cell voltage of only 1.57 V with outstanding long-term durability. This work establishes a new paradigm, demonstrating that rational d-band tuning via crystalline heterointerfaces is a powerful strategy for designing advanced synergistic electrocatalysts.

## 1. Introduction

The production of green hydrogen via water electrolysis is a cornerstone for a sustainable energy future, yet its overall efficiency is largely hampered by the sluggish kinetics of the anodic oxygen evolution reaction (OER) [1,2,3]. Among various candidates, NiFe-based layered double hydroxides (LDHs) have emerged as state-of-the-art earth-abundant electrocatalysts owing to their low cost and excellent intrinsic activity [4,5,6]. However, a paradigm shift in the field now recognizes that these as-prepared materials are merely precatalysts. Under the harsh anodic potentials of OER, they universally undergo dynamic reconstruction to form the true active species, typically amorphous or poorly crystalline metal oxyhydroxides [7,8,9]. Therefore, understanding and ultimately steering this dynamic reconstruction process has become a central theme for the rational design of next-generation OER electrocatalysts.

While dynamic reconstruction is essential for generating highly active catalytic sites [10,11], this uncontrolled process is a double edged sword. Beyond the well-documented issue of cation dissolution, a more insidious degradation pathway is the physical deterioration of the reconstructed active phase [12]. The newly formed, highly dispersed oxyhydroxide nanoclusters are thermodynamically metastable and prone to irreversible agglomeration, coarsening, or amorphization via pathways like Ostwald ripening, leading to a drastic loss of active surface area and eventual catalyst failure [13,14]. Consequently, the scientific challenge has evolved from simply accepting reconstruction to actively guiding it. Developing novel strategies to kinetically trap these highly active, yet transient, nanostructures and arrest their physical degradation is of paramount importance for achieving ultra-durable catalysts.

To address this stability challenge, various interfacial engineering strategies, such as applying protective coatings, have been explored [15,16]. However, a fundamental dilemma often arises: enhancing stability typically comes at the expense of catalytic activity. Conventional protective layers can passivate the catalyst surface, block active sites, or impede charge transfer. An ideal interface should not only act as a physical barrier but also electronically activate the species it protects. Achieving such an activating effect with a chemically inert or even electrically insulating interface is counterintuitive and represents a significant scientific hurdle. This raises a critical question: is it possible to design a single, synergistic interface that simultaneously exerts physical confinement on the active phase to prevent structural degradation and electronically modulates it to boost intrinsic activity?

Recent work by Zhu et al. has shown that liquid-exfoliated boron nitride nanosheets can be assembled on three-dimensional NiFe-LDH electrodes by electrophoretic deposition to improve OER activity and durability [17]. Here, we investigate a crystalline BN interlayer directly deposited on hydrothermally grown FeNi-LDH nanosheet arrays by low-temperature plasma treatment. Operando Raman spectroscopy shows that the BN-modified electrode develops Raman features assigned to NiOOH at a lower potential than the unmodified electrode. XPS measurements and DFT calculations support interfacial charge redistribution and modified electronic states of the Fe and Ni sites. Post-reaction characterization further shows the retention of B-N bonding and the BN-containing heterostructure after electrolysis. The BN-modified electrode requires overpotentials of 234 mV for OER and 98 mV for HER at 10 mA cm^−2^. In a two-electrode configuration, it reaches 10 mA cm^−2^ at 1.57 V and operates at 100 mA cm^−2^ for 60 h. These results indicate that a plasma-grown BN interface can couple reconstructive activation with electronic regulation in FeNi-LDH-based electrodes.

## 2. Results and Discussion

### 2.1. Synthesis and Structural Characterization of BN-FeNi LDH

**Figure 1:**
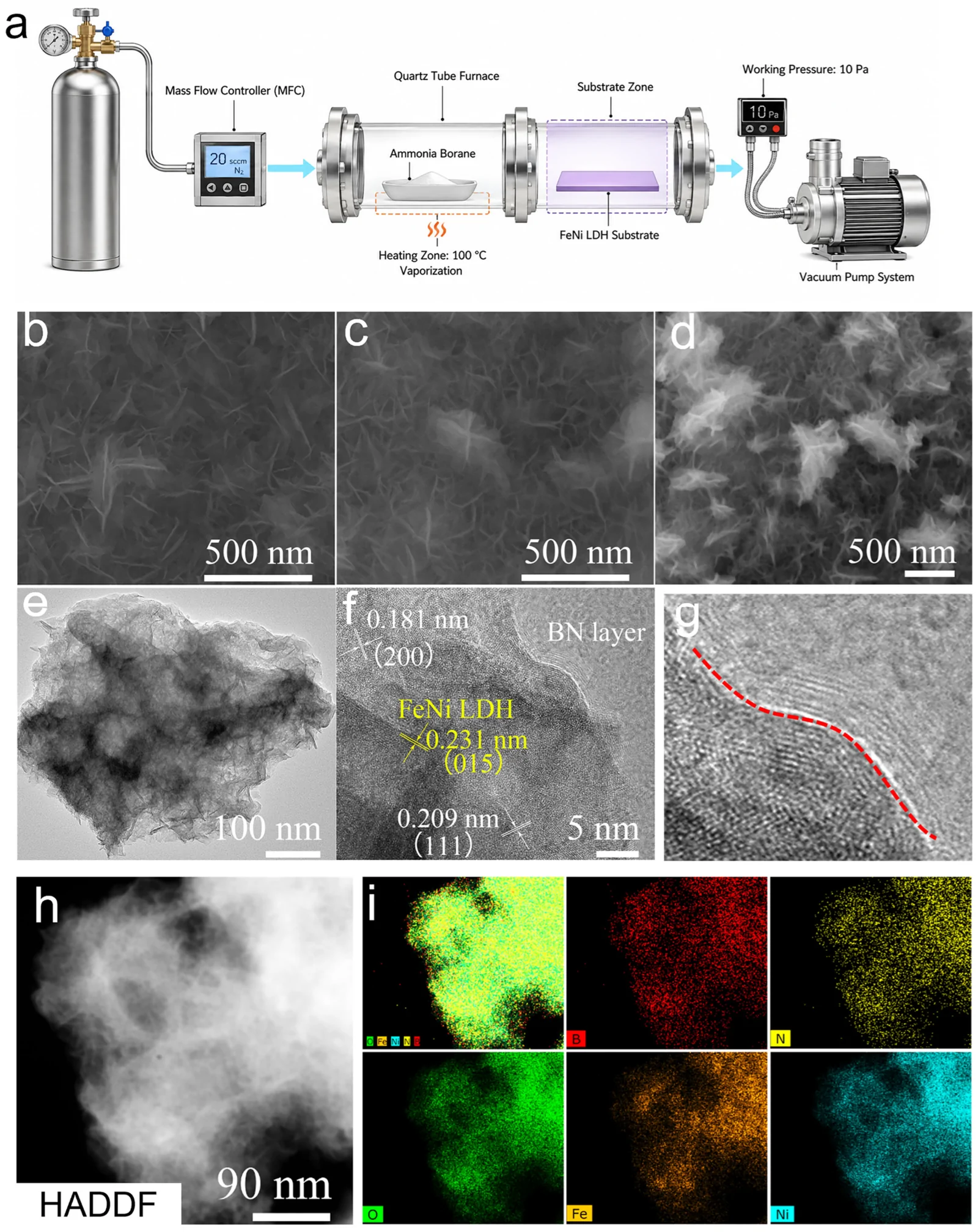
(**a**) Schematic illustration of the BN layer deposition on NiFe LDH via low-temperature plasma using ammonia borane. (**b**–**d**) SEM images of (**b**) pristine FeNi-LDH, (**c**) Ar plasma-treated FeNi-LDH, and (**d**) the optimized BN-FeNi LDH. (**e**–**g**) TEM and HRTEM images of the optimized BN-FeNi LDH. (**h**) STEM image and (**i**) corresponding EDX elemental mapping of the optimized BN-FeNi LDH.

The synthesis of the BN-FeNi LDH heterostructure is illustrated in Figure 1a. The process began with the fabrication of the precursor, a vertically aligned FeNi-LDH nanosheet array on nickel foam (NF), via a hydrothermal method. The high-magnification scanning electron microscopy (SEM) image of the pristine FeNi-LDH (Figure 1b) reveals a highly porous, three-dimensional scaffold composed of interconnected, flake-like nanosheets. This morphology provides an ideal high-surface-area substrate for subsequent interfacial engineering.

A core challenge in such two-step syntheses is to deposit the functional layer without damaging the delicate nanostructure of the substrate. We employed a low-temperature Ar-plasma process to deposit the BN layer from an ammonia borane precursor. To isolate the effects of the plasma treatment itself, we prepared a crucial control sample (Ar-FeNi LDH) by exposing the pristine FeNi-LDH to the Ar plasma for an extended duration without the ammonia borane precursor. As shown in the SEM image (Figure 1c), the morphology of the Ar-FeNi LDH sample is virtually indistinguishable from the pristine FeNi-LDH (Figure 1b and Figure S1). The similar nanosheet morphologies of pristine FeNi-LDH and Ar–FeNi-LDH indicate that Ar plasma treatment under the selected conditions causes no obvious morphological damage (Figure 1b,c). This control reduces the likelihood that the observed performance enhancement arises primarily from plasma-induced morphological changes. The BN deposition time was subsequently optimized by comparing SEM images of samples treated for 10, 20, 40, and 60 min, followed by alkaline washing (Figure S2). The nanosheet framework was retained at shorter deposition times, whereas surface deposits became progressively more pronounced as the treatment time increased. After 60 min, substantial aggregates were observed on the nanosheet surface (Figure S2j–l), indicating excessive deposition. A deposition time of 30 min was therefore selected. The resulting BN–FeNi-LDH retained the open nanosheet architecture while exhibiting a denser, coated surface (Figure 1d).

**Figure 2:**
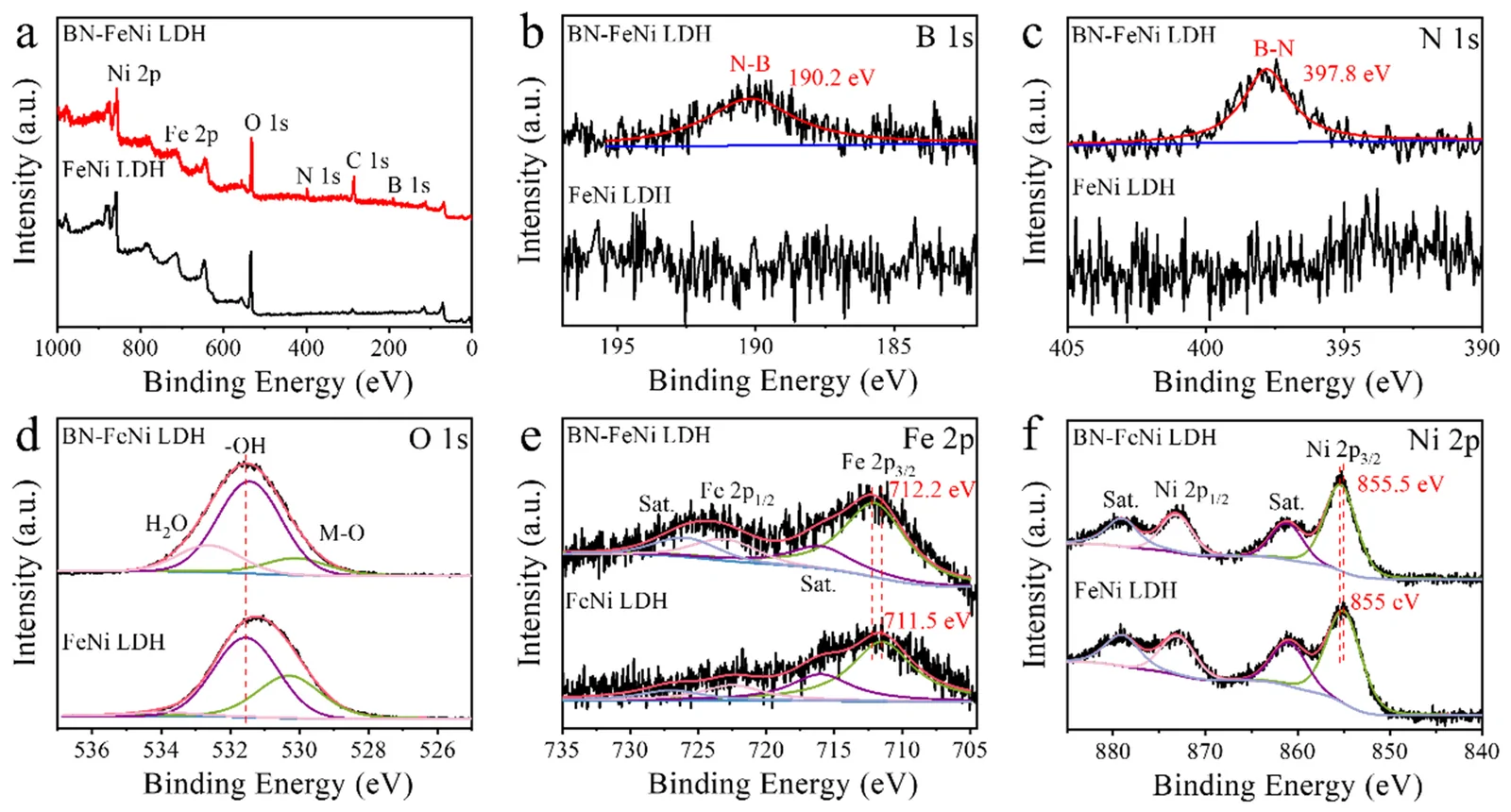
Comparative XPS analysis of FeNi LDH and BN-FeNi LDH. (**a**) Survey spectra. High-resolution core-level spectra of the (**b**) B 1s, (**c**) N 1s, (**d**) O 1s, (**e**) Fe 2p, and (**f**) Ni 2p regions.

The heterostructural nature and elemental distribution were confirmed by transmission electron microscopy (TEM) and energy-dispersive X-ray (EDX) mapping. The low-magnification TEM image (Figure 1e) clearly shows the BN layer conformally coating the nanosheet, preserving the substrate’s morphology. Further analysis by high-resolution TEM (HRTEM) provides definitive evidence of a highly crystalline, core–shell heterostructure (Figure 1f,g). As shown in Figure 1f, lattice fringes with an interplanar spacing of 0.231 nm are observed, corresponding to the (015) plane of the crystalline FeNi-LDH core [18]. Crucially, this core is encapsulated by a multi-layer crystalline BN shell. Figure 1g provides a magnified view of the BN-containing surface region. Lattice-fringe intensity profiles yield interplanar spacings of approximately 0.209 and 0.181 nm. The HRTEM shows that the reciprocal-lattice geometry support assignment of these fringes to the h-BN (101) and (102) planes, respectively. These results indicate that a crystalline h-BN layer is in close contact with the FeNi-LDH substrate and support the use of an h-BN/NiFeO_x_H_γ_ interface in the theoretical model. Furthermore, the EDX elemental mapping (Figure 1h,i and Figure S3) demonstrates a uniform and co-located distribution of B and N signals across the entire Fe, Ni, and O-rich nanosheet, corroborating the formation of a homogeneous BN-FeNi LDH heterostructure. To ensure a chemically pure interface, a facile alkaline wash step was implemented post-synthesis. The efficacy of this purification step and the definitive confirmation of a pure, crystalline B-N bonded interface (free of B-O impurities) will be rigorously proven in the following X-ray photoelectron spectroscopy (XPS) analysis (Figure 2).

Following the structural verification, X-ray photoelectron spectroscopy (XPS) was employed to confirm the chemical purity and, crucially, to probe the electronic interactions at the BN-FeNi LDH heterointerface. The survey spectrum (Figure 2a) confirms the presence of B 1s and N 1s signals in the BN-FeNi LDH sample, while the attenuated Fe 2p and Ni 2p signal intensities, relative to pristine FeNi LDH, are consistent with the surface being conformally coated by the multi-layer BN. We first established the chemical nature and successful deposition of the BN layer. The high-resolution B 1s spectrum (Figure 2b) for the BN-FeNi LDH sample is dominated by a single, deconvoluted peak centered at 190.2 eV, characteristic of B-N bonds [19]. Crucially, this peak is, as expected, entirely absent in the pristine FeNi LDH control sample, confirming the successful deposition of boron. Furthermore, the negligible intensity of characteristic B-O bond signals (typically ~192 eV) confirms the formation of a chemically pure B-N interface rather than a mixed B-N-O [20]. The N 1s spectrum (Figure 2c) further corroborates this conclusion, displaying a corresponding B-N peak at 397.8 eV that is also absent in the control sample. This rigorous verification of a pure B-N interface is essential, allowing us to isolate and study the true electronic effects of this specific interface, rather than a mixed B-N-O.

Having identified B–N bonding in the surface coating, we examined its association with the local electronic environments of Fe and Ni. As shown in Figure 2e, the Fe 2p_3/2_ peak shifts from 711.5 eV for FeNi-LDH to 712.2 eV for BN–FeNi-LDH, corresponding to a positive shift of 0.7 eV [21]. Similarly, the Ni 2p_3/2_ peak shifts from 855.0 to 855.5 eV after BN deposition, giving a positive shift of 0.5 eV (Figure 2f). These shifts indicate changes in the local chemical environments of Fe and Ni following BN deposition. Although shifts toward higher binding energies can be consistent with decreased electron density at the metal centers, they may also be influenced by coordination, oxidation state, surface charging, and final-state effects. Therefore, XPS alone does not establish the direction or magnitude of charge transfer. When considered together with the DFT charge-density-difference results, the XPS data support interfacial electronic redistribution between the BN-containing coating and FeNi-LDH. This modulation, in turn, manifests in the chemical environment of the catalyst surface, as revealed by the O 1s spectra (Figure 2d). It is widely accepted that a higher abundance of surface metal-hydroxyl (M-OH) groups, relative to metal-oxide (M-O) bonds, facilitates the adsorption of OER intermediates and promotes catalysis [22]. Upon deconvolution, the M-OH/M-O ratio for BN-FeNi LDH is significantly higher than that of pristine FeNi LDH. This increase in surface hydroxylation is not an artifact of the synthesis, but rather a direct consequence of the altered electronic structure of the Fe/Ni centers (as shown in Figure 2e–f), which modifies their affinity for adsorbing hydroxyl species.

### 2.2. Electrocatalytic OER Performance and Mechanistic Insights

**Figure 3:**
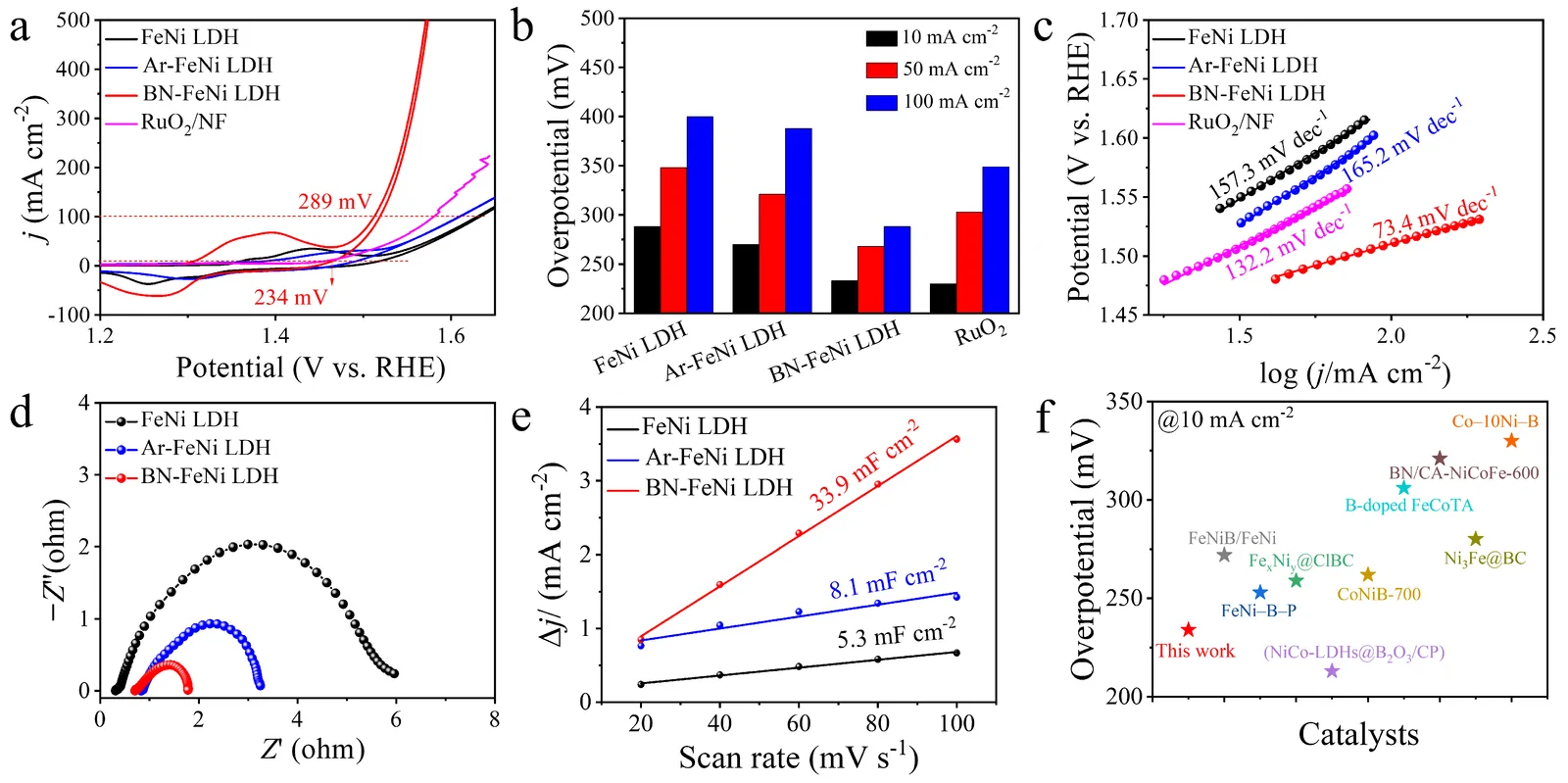
OER electrochemical performance of catalysts in 1 M KOH. (**a**) LSV polarization curves for FeNi LDH, Ar-FeNi LDH, BN-FeNi LDH, and RuO_2_. (**b**) The corresponding Tafel plots. (**c**) Comparison of overpotentials required to achieve current densities of 10, 50, and 100 mA cm^−2^. (**d**) Nyquist plots. (**e**) Capacitive current density differences plotted against scan rates (used for C_dl_ calculations). (**f**) Comparisons of OER performance of different catalysts.

Having established the successful synthesis of a pure, electronically coupled BN-FeNi LDH heterostructure, we next evaluated its electrocatalytic performance for the oxygen evolution reaction (OER). The OER activity was assessed using linear sweep voltammetry (LSV) in 1 M KOH (Figure 3a). The BN-FeNi LDH catalyst exhibits exceptional activity, requiring a low overpotential of only 234 mV to achieve the benchmark current density of 10 mA cm^−2^. This performance markedly surpasses that of pristine FeNi LDH and the benchmark RuO_2_ catalyst. Crucially, the BN-FeNi LDH also dramatically outperforms the Ar-FeNi LDH sample. This control group, which possesses the same plasma-treated physical morphology as our catalyst but lacks the BN-induced electronic modulation, performs similarly poorly to the pristine FeNi LDH. This finding is a critical piece of evidence: it definitively proves that the observed catalytic enhancement is not an artifact of plasma-induced damage or defect engineering, but rather a direct consequence of the BN heterointerface itself. The superior performance of BN-FeNi LDH is maintained at high current densities, delivering 50 mA cm^−2^ and 100 mA cm^−2^ at low overpotentials of 268 mV and 289 mV, respectively (Figure 3b), highlighting its potential for industrial applications.

To probe the reaction kinetics, Tafel slopes were derived from the LSV curves (Figure 3c). The BN-FeNi LDH displays a small Tafel slope of 73.4 mV dec^−1^, indicating highly favorable OER kinetics. In stark contrast, both the pristine FeNi LDH (157.3 mV dec^−1^) and the Ar-FeNi LDH (165.2 mV dec^−1^) exhibit significantly larger slopes. This stark disparity in kinetics clearly distinguishes the origin of the enhancement, attributing the accelerated OER kinetics solely to the chemical/electronic effects of the BN interface, not the plasma process itself. The sluggish kinetics of the control samples are further corroborated by electrochemical impedance spectroscopy (EIS) (Figure 3d). The BN-FeNi LDH catalyst exhibits the smallest Nyquist plot semicircle, corresponding to the lowest charge transfer resistance [23]. This rapid charge transfer confirms the superior kinetic pathway established by the BN interface, which is consistent with the XPS findings (Figure 2) of a more favorable, hydroxyl-rich surface environment. To deconvolve the contributions of active site quantity versus intrinsic quality, the electrochemical active surface area (ECSA) was estimated via the double-layer capacitance (C_dl_) (Figure 3e and Figure S4) [24]. The C_dl_ for BN-FeNi LDH (33.9 mF cm^−2^) is dramatically larger than that of both FeNi LDH (5.3 mF cm^−2^) and the Ar-FeNi LDH sample (8.1 mF cm^−2^). This ~6.4-fold increase in ECSA suggests the BN interface significantly increases the number of electrochemically accessible active sites, likely by facilitating the surface hydroxylation (as seen in O 1s spectra) and activating previously dormant regions.

This massive increase in double-layer capacitance translates to a dramatically enhanced ECSA. The calculated ECSA for BN-FeNi LDH is 847.5 cm^2^, a value approximately 6.4-fold and 4.2-fold larger than that of pristine FeNi LDH (132.5 cm^2^) and the Ar-FeNi LDH sample (202.5 cm^2^), respectively. This dramatically enhanced ECSA, however, does not fully account for the dramatic performance boost. To isolate the intrinsic activity, we calculated the ECSA normalized specific activity. Even after accounting for its vastly larger active area, the BN-FeNi LDH catalyst still exhibits the highest specific activity at a given overpotential. This is the definitive proof of our core hypothesis: the electronic modulation from the BN layer (Figure 2) achieves a dual enhancement, simultaneously increasing the quantity of accessible sites and boosting their intrinsic catalytic turnover rate. Finally, when benchmarked against recently reported B or N-containing non-noble metal OER catalysts (Figure 3f), our BN-FeNi LDH catalyst is positioned among the most active materials reported to date [19,25,26,27,28,29,30,31,32].

### 2.3. In Situ Raman Spectroscopy: Probing the Origin of Activity

**Figure 4:**
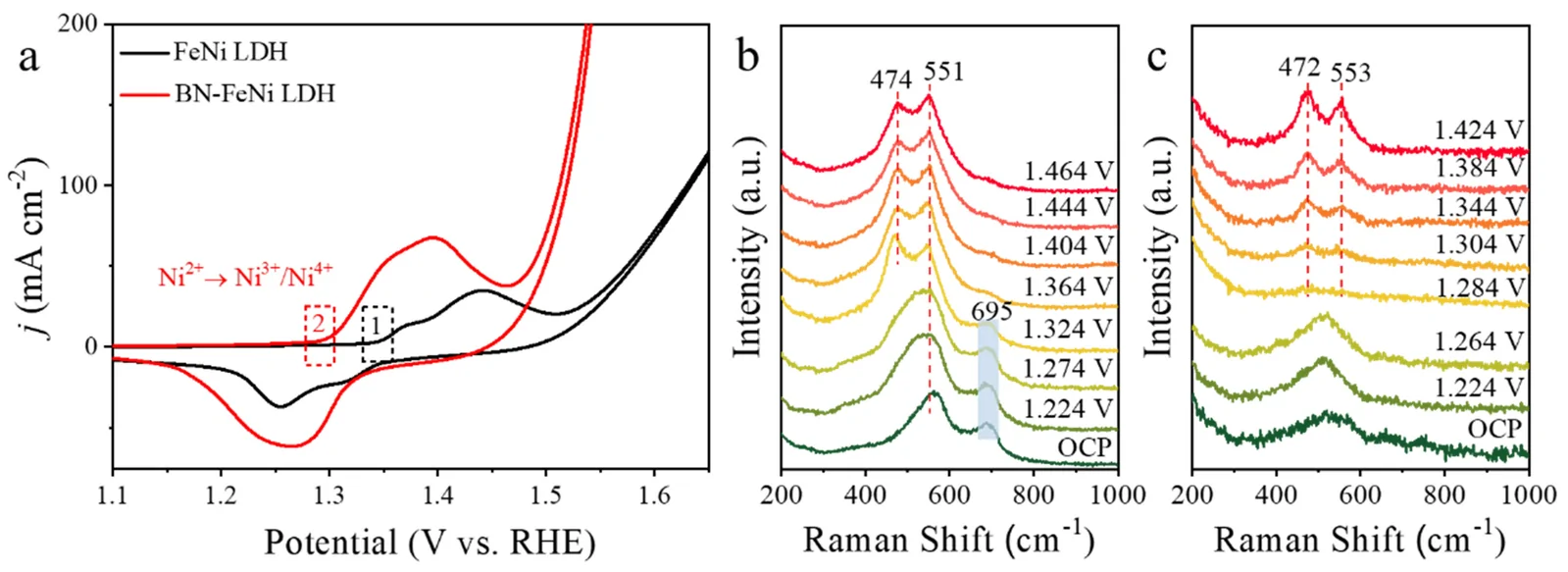
Cyclic-voltammetry curves of FeNi LDH and BN-FeNi LDH. (**b**,**c**) In situ Raman spectra of FeNi LDH and BN-FeNi LDH. (**a**) LSV curves of FeNi LDH (black) and BN-FeNi LDH (red) in 1 M KOH. The dashed boxes 1 and 2 indicate the potential regions corresponding to the Ni2+→Ni3+/Ni4+ oxidation process. Compared with FeNi LDH, BN-FeNi LDH exhibits a negatively shifted oxidation peak.

To elucidate the dynamic evolution of the active phase and understand the origin of the enhanced activity, in situ Raman spectroscopy was employed to track the structural changes in FeNi LDH and BN-FeNi LDH under applied OER potentials. First, the cyclic voltammetry (CV) curves in Figure 4a provide the electrochemical context. Both catalysts display a distinct anodic peak attributed to the Ni^2+^ to Ni^3+^/Ni^4+^ oxidation, which represents the formation of the true NiOOH active phase from the LDH precatalyst [33]. Critically, this oxidation peak for BN-FeNi LDH (labeled Area 2) occurs at a significantly lower potential than for pristine FeNi LDH, providing the first electrochemical hint of a lowered energetic barrier for reconstruction. The in situ Raman spectra provide complementary evidence for this potential-dependent transformation. At open-circuit potential (OPC), FeNi-LDH exhibits broad bands at 551 and 695 cm^−1^, assigned to Ni–O and Fe–O vibrations of the LDH precursor, respectively [30,34]. As the potential is anodically swept, these precursor peaks remain until a potential of 1.324 V (vs. RHE) is surpassed. At this point, two new, strong bands emerge at 474 cm^−1^ (δ, bending) and 551 cm^−1^ (ν, stretching), which are the well-established signatures of the catalytically active NiOOH phase [35]. This onset potential for reconstruction (1.324 V) correlates perfectly with the electrochemical oxidation peak observed in its CV (Figure 4a). A dramatically different behavior is observed for BN-FeNi LDH (Figure 4c). While the initial OCP peaks are weak (likely attenuated by the BN overlayer), the emergence of the identical NiOOH active phase peaks (at 472 and 553 cm^−1^) occurs at a potential of only 1.284 V (vs. RHE). This represents a 40 mV negative shift in the formation potential of the true active phase compared to the pristine catalyst. This 40 mV difference is a critical finding. It is the direct spectroscopic manifestation of the electronic modulation proven by XPS (Figure 2). The interfacial charge transfer from FeNi LDH to the BN layer makes the Ni centers more electron deficient, thereby lowering the thermodynamic energy barrier required to oxidize Ni^2+^ to the active Ni^3+^ state. The BN layer, therefore, does not simply act as a protective coating; it functions as an electronic promoter that accelerates the formation of the catalytically active phase. Furthermore, a subtle but important difference in the evolution of the active phase is noted. For pristine FeNi LDH, the peak ratio of the 551 cm^−1^ band gradually overtakes the 474 cm^−1^ band, suggesting an evolving or heterogeneous active structure. In sharp contrast, for BN-FeNi LDH, the intensity ratio of the 472 and 553 cm^−1^ peaks remains constant once formed. This implies that the BN interface not only facilitates an earlier reconstruction but also stabilizes a more uniform and consistent active phase throughout the entire OER process, which is consistent with the enhanced stability observed.

### 2.4. Dual-Functional HER and Overall Water Splitting Performance

**Figure 5:**
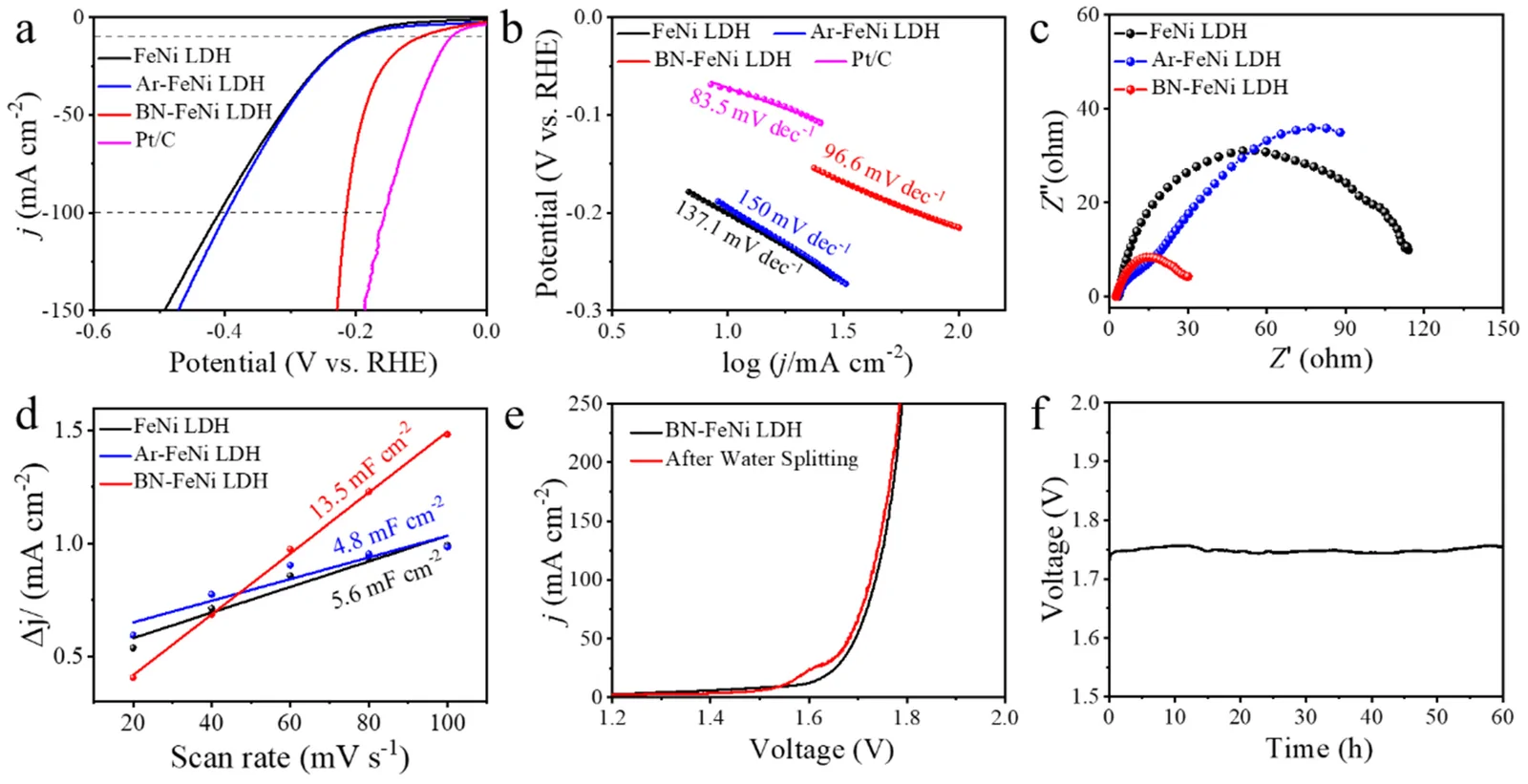
HER and Overall Water Splitting Performance of Catalysts in 1 M KOH. (**a**) HER LSV polarization curves and (**b**) corresponding Tafel plots for FeNi LDH, Ar-FeNi LDH, BN-FeNi LDH, and Pt/C. (**c**) Nyquist plots for FeNi LDH, Ar-FeNi LDH, and BN-FeNi LDH. (**d**) Capacitive current density differences (Δj) plotted against scan rates for ECSA determination. (**e**) LSV curves for overall water splitting in a two-electrode setup, recorded before and after the stability test. (**f**) Chronopotentiometric response of the BN–FeNi-LDH||BN–FeNi-LDH electrolyzer at 10 mA cm^−2^ for 60 h in 1.0 M KOH.

Given the profound electronic modulation imparted by the BN interface, we sought to determine if this effect was exclusive to OER or if it extended to the hydrogen evolution reaction (HER). As shown in Figure 5a, the BN-FeNi LDH exhibits outstanding HER activity, requiring a low overpotential of only 98 mV to reach −10 mA cm^−2^. Critically, this performance is not only superior to the pristine FeNi LDH but also dramatically exceeds the Ar-FeNi LDH sample, which performs almost identically to the pristine catalyst. This result is a key finding: it confirms that the enhancement mechanism is not a physical artifact of the plasma process but is a direct consequence of the BN electronic interface. This superior activity is rooted in accelerated kinetics. The Tafel slope for BN-FeNi LDH (Figure 5b) is 96.6 mV dec^−1^, significantly smaller than that of FeNi LDH (137.1 mV dec^−1^) and the Ar-FeNi LDH (150 mV dec^−1^). This kinetic advantage is further corroborated by EIS (Figure 5c), where BN-FeNi LDH displays the smallest charge transfer resistance, confirming rapid HER kinetics.

To deconvolve the origins of this HER enhancement, we again analyzed the ECSA via the C_dl_ (Figure 5d and Figure S5). The C_dl_ for BN-FeNi LDH (13.5 mF cm^−2^) is approximately 2.4-fold and 2.8-fold larger than for FeNi LDH (5.6 mF cm^−2^) and Ar-FeNi LDH (4.8 mF cm^−2^), respectively. This indicates that, just as with OER, the BN interface creates a dual enhancement for HER by simultaneously increasing the quantity of accessible active sites while also boosting their intrinsic quality. The demonstrated excellence as a bifunctional catalyst prompted the assembly of a two-electrode electrolyzer, using BN-FeNi LDH as both the anode and cathode (Figure 5e). The device exhibits exceptional performance, achieving the 10 mA cm^−2^ benchmark at a cell voltage of only 1.57 V. This voltage, corresponding to a combined overpotential of just 340 mV, positions this catalyst among the most efficient NiFe-based bifunctional systems reported [36].

Finally, the practical durability was confirmed via a long-term chronopotentiometry test at a high current density of 100 mA cm^−2^ (Figure 5f). The electrolyzer shows negligible voltage degradation over 60 h, underscoring its outstanding operational stability. This exceptional durability is rationalized by extensive post-mortem characterization. After stability test of OER, the morphology is altered, but TEM confirms the BN overlayer remains intact on the underlying structure. Figures S6 and S7 show that the interconnected nanosheet framework is largely retained, although the lattice contrast becomes less uniform and short-range crystalline domains appear within a more disordered matrix (Figures S6 and S7). No obvious newly formed large particles are observed in the examined regions. The HER cathode also retains its nanosheet-like morphology after testing (Figures S8 and S9). These ex situ observations indicate morphological retention and limited severe coarsening, but they do not directly establish continuous physical confinement by the BN coating. Crucially, post-stability XPS (Figure S10) confirms the persistent presence of B-N bonds and the sustained positive shift in Fe/Ni binding energies. This confirms that the BN layer acts as a robust physical and chemical shield that locks in the electronically modulated active structure, preventing degradation and ensuring long-term, high-performance operation.

### 2.5. Theoretical Insights into the OER Enhancement Mechanism

**Figure 6:**
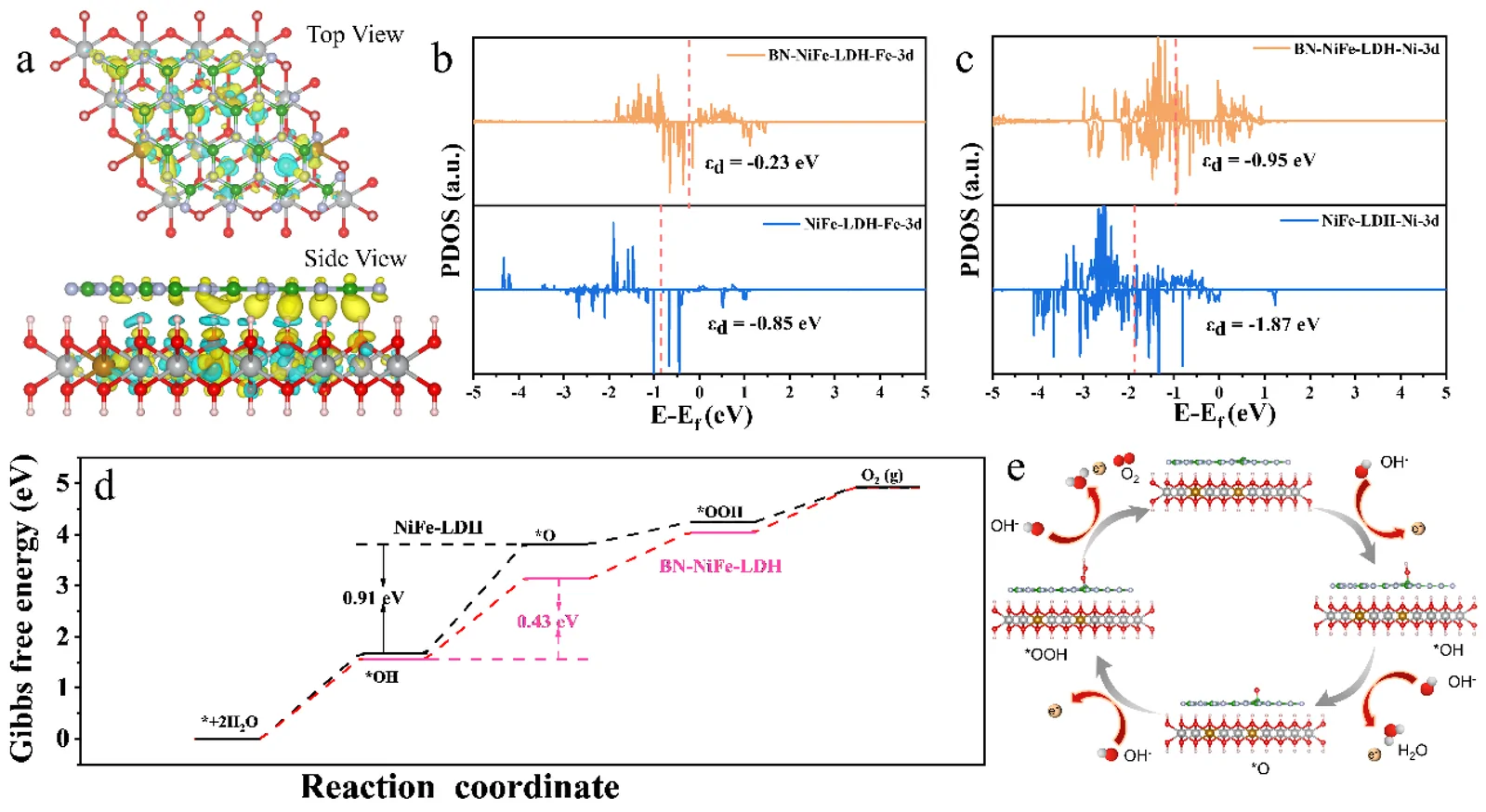
Theoretical Insights into the Electronic Modulation and OER Enhancement Mechanism. (**a**) Calculated charge density difference plot for the BN-FeNi LDH heterostructure, illustrating interfacial charge transfer (cyan and yellow regions represent electron depletion and accumulation, respectively). (**b**,**c**) Projected Density of States (PDOS) of the (**b**) Fe 3d and (**c**) Ni 3d orbitals for pristine FeNi LDH and BN-FeNi LDH, indicating the upward shift in the d-band center upon BN interfacing. (**d**) Calculated Gibbs free energy diagram for the four-electron OER pathway on FeNi LDH and BN-FeNi LDH. (**e**) Schematic illustration of the OER mechanism on the electronically modulated BN-FeNi LDH interface. (* represents adsorbed species).

We used density functional theory calculations to examine how the BN interface affects the electronic structure and OER thermodynamics of the reconstructed NiFeO_x_H_γ_ active phase. ICP-OES measurements gave an experimental Ni/Fe atomic ratio of 2.67:1. We therefore used a periodic NiFeO_x_H_γ_ model with a Ni/Fe ratio of 3:1 as an approximate representation of the measured composition. The h-BN layer in the model represents the BN layer directly contacting the oxyhydroxide surface. The optimized structures and detailed atomic configurations of NiFeO_x_H_γ_ and h-BN/NiFeO_x_H_γ_ are shown in Figure S11. The charge-density-difference map in Figure 6a shows electron accumulation on the h-BN layer and electron depletion around the adjacent Fe and Ni sites of NiFeO_x_H_γ_. This calculated redistribution is consistent with the direction of interfacial charge transfer suggested by the Fe and Ni core-level shifts in the XPS spectra. It does not, by itself, constitute direct experimental proof of charge transfer. This electronic pull effect has a profound and quantifiable impact on the d-electron configuration of the metal active centers. We analyzed the projected density of states (PDOS) to calculate the d-band center (ε_d_), a key descriptor for catalytic activity. For the pristine FeNi LDH model, the ε_d_ for the active Fe and Ni sites were calculated to be −0.85 eV and −1.87 eV, respectively. Remarkably, upon interfacing with the BN layer (Figure 6b,c), the ε_d_ for both the Fe site and the Ni site shifts significantly upward (closer to the Fermi level) to −0.23 eV and −0.95 eV, respectively. According to d-band theory, this upward shift in ε_d_ strengthens the chemisorption of OER intermediates. To determine how this modulation affects the reaction, we calculated the Gibbs free energy (∆G) diagram for the entire four-electron OER pathway (Figure 6d). For pristine FeNi LDH, the deprotonation of *OH to form *O (*OH → *O) was identified as the potential-determining step (PDS), presenting a formidable energy barrier of 0.91 eV. Crucially, on the BN-FeNi LDH model, this exact same step is no longer the bottleneck; the barrier is dramatically reduced to just 0.43 eV. Furthermore, the ∆G for all intermediate steps on the BN-FeNi LDH surface are systematically lower than on the pristine counterpart. This theoretical analysis provides a complete and elegant explanation for the enhanced performance. The electronic pull from the BN layer upshifts the d-band centers of the Fe and Ni active sites. This electronic modulation optimizes the adsorption energies of all OER intermediates, but specifically breaks the unfavorable scaling relationship on the pristine surface by dramatically lowering the energy barrier of the PDS. This theoretical finding perfectly explains the accelerated kinetics (Figure 3b) and lowered overpotential (Figure 3a) observed experimentally, with the entire optimized process summarized schematically in Figure 6e.

## 3. Materials and Methods

### 3.1. Chemicals and Materials

Borane–ammonia complex (97%), Iron (III) nitrate nonahydrate (Fe(NO_3_)_3_·9H_2_O, AR, 97%), nickel (II) nitrate hexahydrate (Ni(NO_3_)_2_·6H_2_O, AR, 98.5%) obtained from Aladdin. Ethanol (99.7%) was purchased from Kelong Chemical Company, Chengdu, China. All chemicals were used as received, without further purification.

### 3.2. Synthesis of Electrocatalysts

A piece of nickel foam (NF, 40 × 10 × 1.8 mm) was utilized as the substrate. The NF was pre-cleaned by ultrasonication, sequentially, in 0.5 M hydrochloric acid, 95% ethanol, and deionized water. The cleaned NF was subsequently dried at 60 °C. For the hydrothermal synthesis, 30 mL of deionized water was added to a 50 mL reaction vessel. Next, 1.0 mL of 0.5 M iron nitrate solution and 1.0 mL of 0.5 M nickel nitrate solution were added to the water and mixed evenly to form the precursor solution. The pre-cleaned NF was then immersed in this mixed solution. The vessel was sealed and placed in an oven, where it was heated at 160 °C for 12 h. After the reaction, the vessel was allowed to cool to room temperature. The resulting FeNi LDH/NF sample was retrieved, washed three times with deionized water, and subsequently dried.

The BN-FeNi LDH samples were prepared via a plasma treatment process. A FeNi LDH substrate (2 cm × 2 cm) was placed inside the plasma chamber. Ammonia borane (50 mg), serving as the BN precursor, was loaded into the front end of the quartz tube. The chamber was then purged with argon gas, and the working pressure was adjusted to 10 Pa.

Prior to deposition, the ammonia borane was pretreated by heating at 70 °C for 2 h to remove adsorbed moisture and impurities. Subsequently, the ammonia borane was heated to 100 °C to generate precursor vapor. The plasma was then ignited and maintained at a power of 200 W and maintained for 60 min. Unless otherwise specified, BN-FeNi-LDH/NF refers to the sample treated for 30 min. After deposition, the sample was cooled under Ar, washed with ethanol for 5 min, rinsed with deionized water, and dried at 60 °C.

For the control experiments, Ar–FeNi-LDH/NF was prepared under the same plasma power, Ar flow rate, pressure, treatment time, and post-treatment conditions, but without ammonia borane. BN/NF was prepared by applying the same BN deposition procedure to bare NF. All FeNi-LDH-based samples used for comparison were obtained from the same hydrothermal batch.

### 3.3. Electrochemical Measurements

All electrochemical tests were conducted at room temperature in a 1 M KOH electrolyte using a CHI760e electrochemical workstation (CH Instruments, Inc., Austin, TX, USA). The OER and HER activities of the catalysts were evaluated in a three-electrode cell configuration. The as-prepared electrocatalysts were used as the working electrode, a graphite rod served as the counter electrode, and a Hg/HgO (in 1 M KOH) electrode was used as the reference. All measured potentials were referenced to the reversible hydrogen electrode (RHE) scale according to the equation: E_(RHE)_ = E_(Hg/HgO)_ + 0.098 V + 0.059 × pH. Before polarization measurements, each electrode was activated by 20 CV cycles between at 50 mV s^−1^ until successive curves overlapped. LSV measurements were then conducted at 5 mV s^−1^. The electrochemical characterizations included cyclic voltammetry (CV) and linear sweep voltammetry (LSV) with 90% iR compensation. Additionally, the overall water splitting activity of the catalysts was assessed in a two-electrode cell.

### 3.4. Density Functional Theory (DFT) Calculations

All spin-polarized DFT calculations were performed using the Vienna Ab initio Simulation Package (VASP) [37]. A slab model of the hBN/NiFeOxHy heterostructure, featuring a Ni/Fe ratio of 3:1, was constructed to represent the catalyst. The projector augmented-wave (PAW) method was employed to describe the electron–ion interactions. The exchange-correlation functional was described by the Perdew–Burke–Ernzerhof (PBE) functional within the generalized gradient approximation (GGA) [38]. The DFT-D3 correction was included to account for van der Waals (vdW) interactions [39]. A kinetic energy cutoff of 500 eV was set for the plane-wave basis set. The Brillouin zone was sampled using a 2 × 2 × 1 Γ-centered k-point mesh for geometric optimization. The electronic self-consistency loop was converged with an energy threshold of 10^−5^ eV. The equilibrium geometries and lattice constants were fully optimized until the maximum force (stress) on each atom was less than 0.02 eV Å^−2^. To describe the strong on-site correlations of the Fe and Ni 3d orbitals, the DFT+U method was applied, with effective Hubbard U values of U_Fe_ = 5.3 eV and U_Ni_ = 6.6 eV. All calculations were spin-polarized to model the antiferromagnetic (AFM) state of the hBN/NiFeOxHy and pristine NiFeOxHy systems. During the structural optimization of the hBN/NiFeOxHy heterostructure, the initial magnetic moments were set to 3μ_B_ for half of the Fe atoms and 1μ_B_ for half of the Ni atoms; the moments for the other half of the Fe and Ni atoms were set to −3 μ_B_ and −1 μ_B_, respectively.

### 3.5. Characterizations

The surface morphology and structure were characterized by a scanning electron microscopy (FESEM, thermo scientific Apreo 2C, Thermo Fisher Scientific, Waltham, MA, USA) and a Transmission electron microscopy (TEM, FEI Talos F200x, FEI Company, Hillsboro, OR, USA)). X-ray photoelectron spectroscopy (XPS) (PHI-5000) (Physical Electronics, Chanhassen, MN, USA) using a mono-chromatic Al Kα radiation at 1486.6 eV. Raman spectroscopy was recorded on a LabRAM HR Evolution Raman spectrometer from Horiba Jobin Yvon and the excitation light used is a 532 nm laser.

## 4. Conclusions

In conclusion, this work has designed and validated a synergistic confine and activate strategy, realized in a chemically pure, crystalline BN-FeNi LDH heterostructure, which successfully resolves the persistent activity and stability trade-off in bifunctional water splitting. The catalyst demonstrates outstanding OER and HER performance, enabling high efficiency overall water splitting at a level competitive with elite NiFe-based systems. More profoundly than the performance metrics, our study provides a rigorous, decoupled understanding of the enhancement mechanism. Through critical control experiments, we unequivocally demonstrate that the catalytic gains stem solely from the electronic and chemical contributions of the BN interface, not from physical plasma-induced artifacts. We establish that this interface functions as a powerful electronic modulator, inducing a significant upward shift in the active sites’ d-band center via interfacial charge transfer, a finding corroborated by both XPS and DFT. The true significance of this mechanism lies in its unifying nature. We have demonstrated how this single, fundamental electronic perturbation of the d-band center upshift simultaneously and synergistically optimizes the distinct catalytic pathways of both OER and HER. This modulation breaks the unfavorable scaling relationships for OER by lowering the energy barrier for the rate-determining step, while concurrently tuning the hydrogen adsorption free energy toward the thermoneutral ideal for HER. This study thus provides a strategy for interfacial regulation by illustrating how materials traditionally utilized for passivation can be intelligently engineered into functional electronic layers, thereby opening a novel and rational avenue for designing ultra-stable bifunctional catalysts by simultaneously achieving physical confinement and electronic activation.

## Data Availability

The data presented in this study are available on request from the corresponding authors.

## Supplementary Materials

The following supporting information can be downloaded at: https://www.mdpi.com/article/10.3390/nano16201280/s1, Figure S1. SEM images of FeNi LDH treated with Ar plasma for 60 min without ammonia borane. Figure S2. SEM images of FeNi LDH after BN deposition for different times followed by alkaline washing: (a–c) 10 min, (d–f) 20 min, (g–i) 40 min, and (j–l) 60 min. Figure S3. SEM-EDS elemental mapping images of BN-FeNi LDH. Figure S4. Cyclic voltammograms of FeNi LDH, Ar-FeNi LDH, and BN-FeNi LDH collected at scan rates from 20 to 100 mV s^−1^ for OER Cdl analysis. Figure S5. Cyclic voltammograms of FeNi LDH, Ar-FeNi LDH, and BN-FeNi LDH collected at scan rates from 20 to 100 mV s^−1^ for HER C_dl_ analysis. Figure S6. SEM images of the BN-FeNi LDH electrode after the OER stability test. Figure S7. TEM images of the BN-FeNi LDH electrode after the OER stability test. Figure S8. SEM images of the BN-FeNi LDH electrode after the HER stability test. Figure S9. TEM images of the BN-FeNi LDH electrode after the HER stability test. Figure S10. XPS spectra of BN-FeNi LDH before and after the stability test: (a) survey, (b) B 1s, (c) N 1s, (d) O 1s, (e) Fe 2p, and (f) Ni 2p. Figure S11. Top and side views of the structural models used in the DFT calculations: (a, c) pristine NiFeO_x_H_γ_; and (b, d) the hBN/NiFeO_x_H_γ_; heterostructure. The dashed rectangles indicate the computational unit cells, and the atomic species are identified by the color key.
