# Supplementary material for: Boron Nitride Interfacial Confinement of FeNi Layered Double Hydroxide Reconstruction for Durable Overall Water Splitting
Source: Nanomaterials (Basel). 2026 Oct 9;16(20):1280. doi: 10.3390/nano16201280 (PMC13707506; doi:10.3390/nano16201280)
Supplement: Supplementary file 1 [file nanomaterials-16-01280-s001.zip › nanomaterials-4600982-supplementary.pdf]

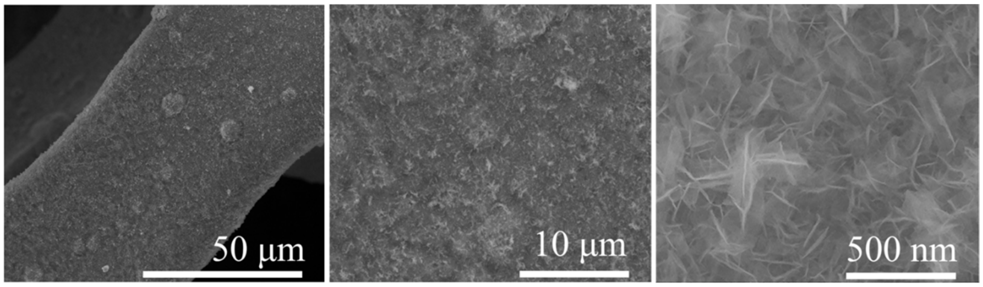

Figure S1. SEM images of FeNi LDH treated with Ar plasma for 60 min without ammonia borane.

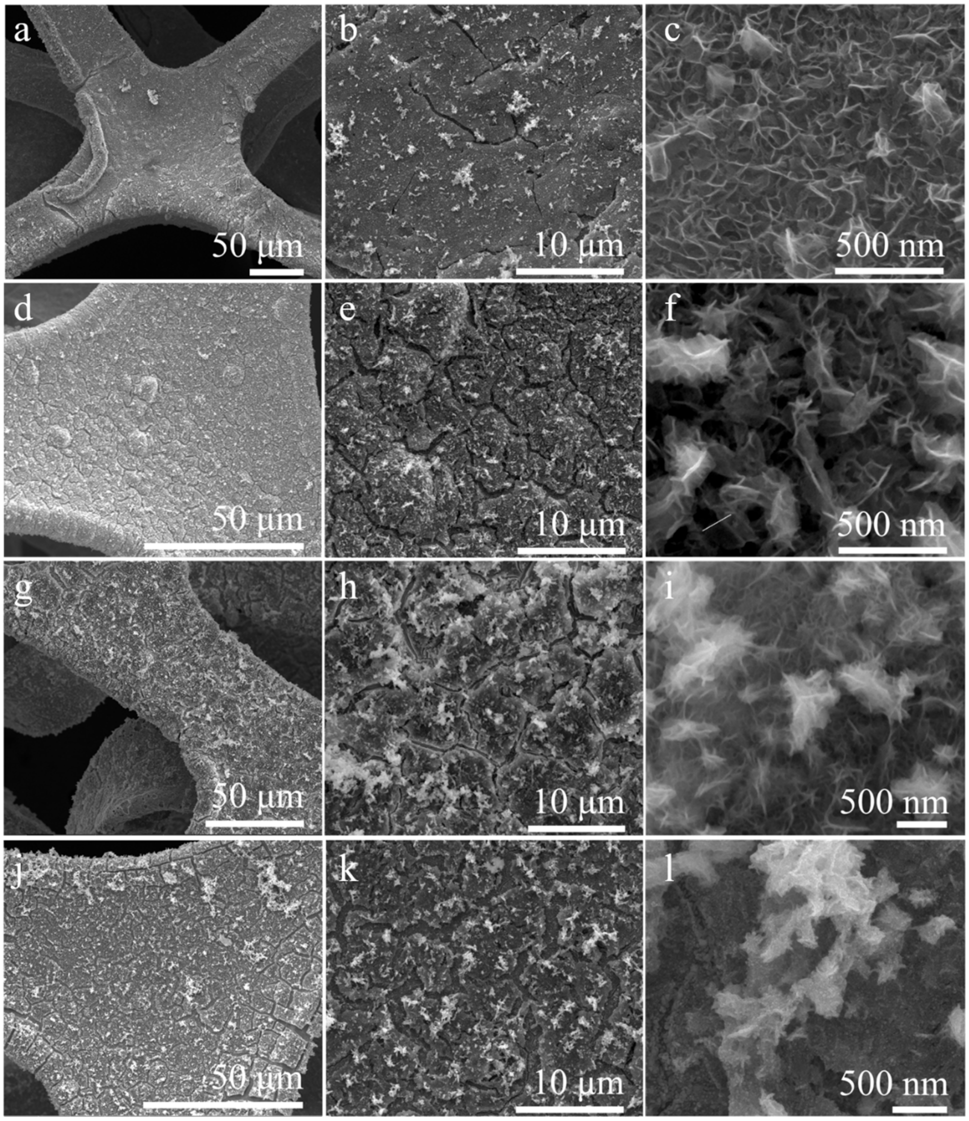

Figure S2. SEM images of FeNi LDH after BN deposition for different times followed by alkaline washing: (a–c) 10 min, (d–f) 20 min, (g–i) 40 min, and (j–l) 60 min.

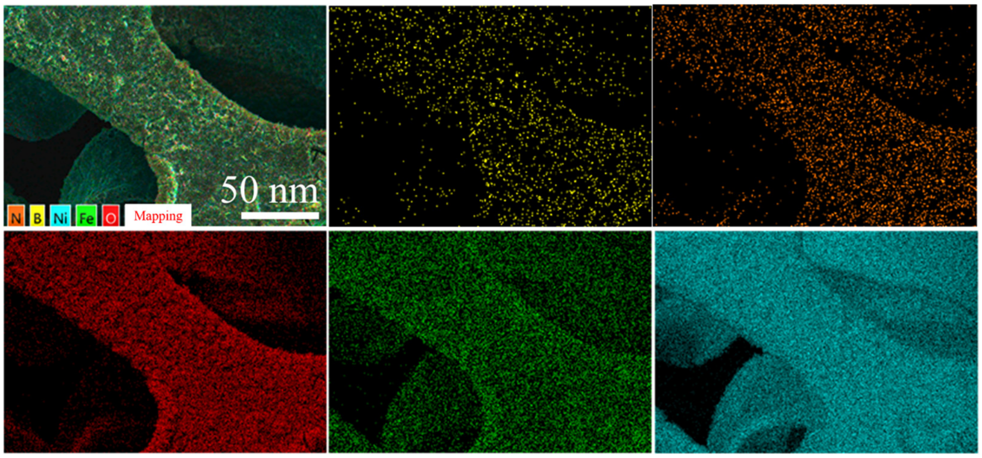

Figure S3. SEM-EDS elemental mapping images of BN-FeNi LDH.

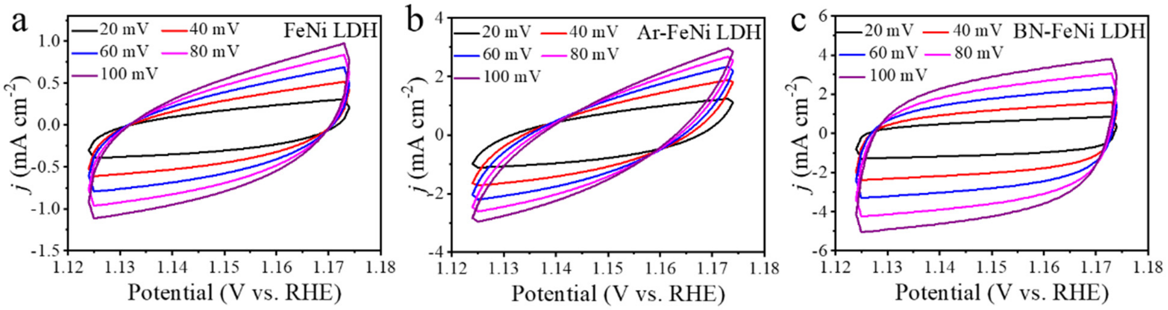

Figure S4. Cyclic voltammograms of FeNi LDH, Ar-FeNi LDH, and BN-FeNi LDH collected at scan rates from 20 to 100 mV s^{-1} for OER C_{dl} analysis.

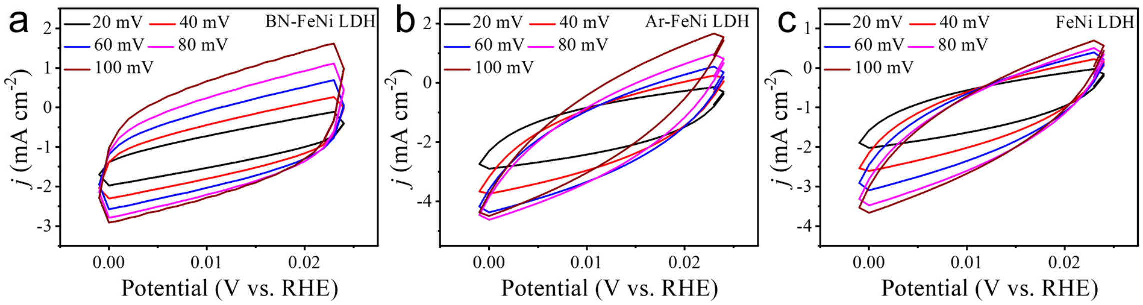

Figure S5. Cyclic voltammograms of FeNi LDH, Ar-FeNi LDH, and BN-FeNi LDH collected at scan rates from 20 to 100 mV s^{-1} for HER C_{dl} analysis.

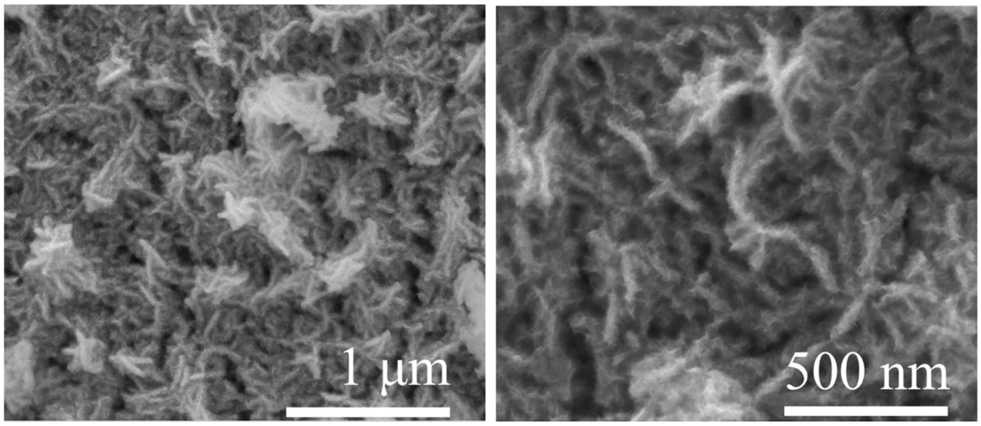

Figure S6. SEM images of the BN-FeNi LDH electrode after the OER stability test.

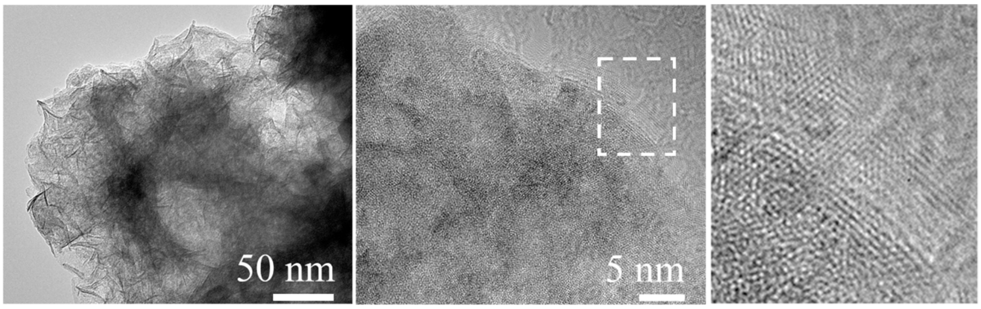

Figure S7. TEM images of the BN-FeNi LDH electrode after the OER stability test.

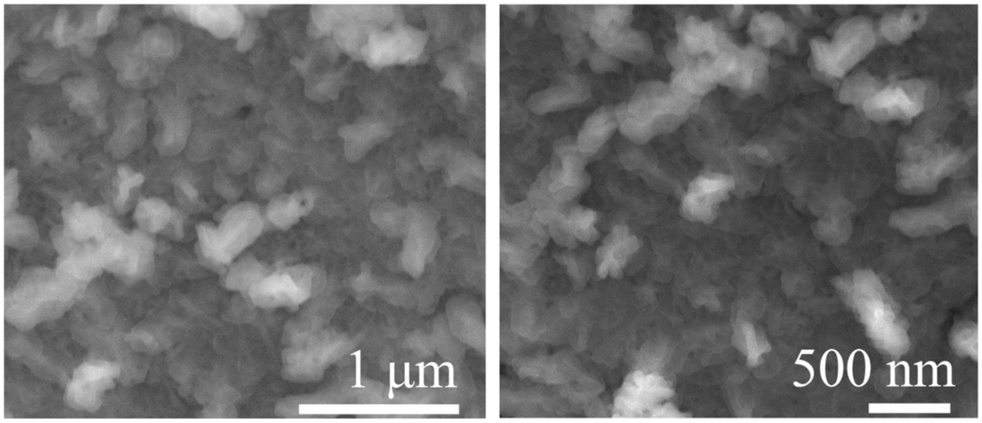

Figure S8. SEM images of the BN-FeNi LDH electrode after the HER stability test.

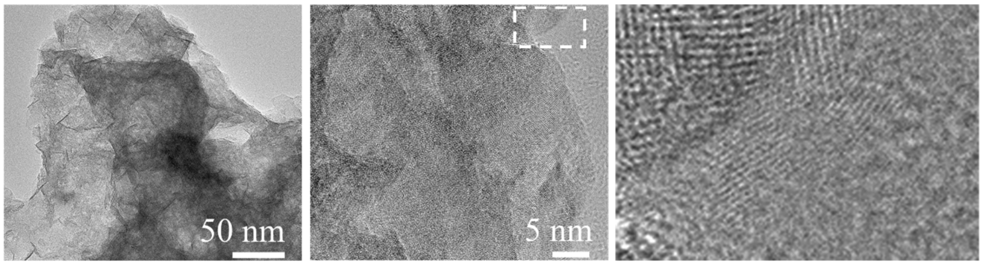

Figure S9. TEM images of the BN-FeNi LDH electrode after the HER stability test.

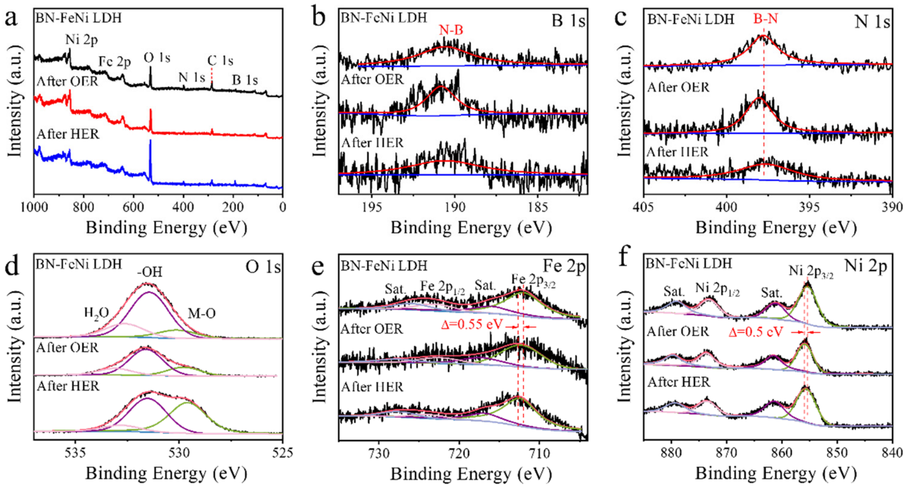

Figure S10. XPS spectra of BN-FeNi LDH before and after the stability test: (a) survey, (b) B 1s, (c) N 1s, (d) O 1s, (e) Fe 2p, and (f) Ni 2p.

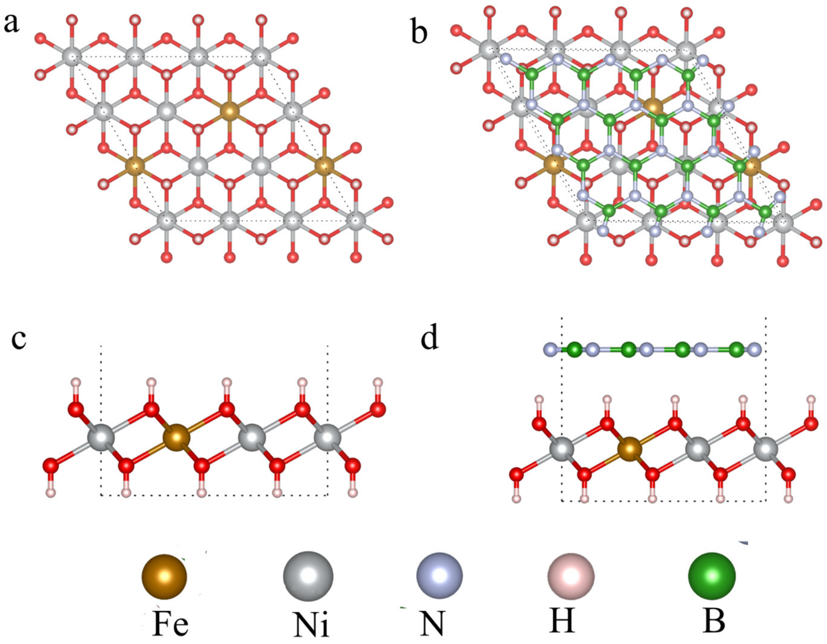

Figure S11. Top and side views of the structural models used in the DFT calculations: (a,c) pristine $NiFeO_xH_Y$ and (b,d) the $hBN/NiFeO_xH_Y$ heterostructure. The dashed rectangles indicate the computational unit cells, and the atomic species are identified by the color key.
